# Supplementary material for: Brain network reconfiguration for narrative and argumentative thought
Source: Commun Biol. 2021 Jun 16;4:746. doi: 10.1038/s42003-021-02151-9 (PMC8208997; doi:10.1038/s42003-021-02151-9)
Supplement: Supplementary file 3 — Reporting Summary [file 42003_2021_2151_MOESM3_ESM.pdf]

nature research

Corresponding author(s): Yangwen Xu

Last updated by author(s): Apr 6, 2021

Reporting Summary

Nature Research wishes to improve the reproducibility of the work that we publish. This form provides structure for consistency and transparency in reporting. For further information on Nature Research policies, see our Editorial Policies and the Editorial Policy Checklist.

Statistics

For all statistical analyses, confirm that the following items are present in the figure legend, table legend, main text, or Methods section.

n/a	Confirmed	
[ ]	[x]	The exact sample size (*n*) for each experimental group/condition, given as a discrete number and unit of measurement
[ ]	[x]	A statement on whether measurements were taken from distinct samples or whether the same sample was measured repeatedly
[ ]	[x]	The statistical test(s) used AND whether they are one- or two-sided<br>*Only common tests should be described solely by name; describe more complex techniques in the Methods section.*
[ ]	[x]	A description of all covariates tested
[ ]	[x]	A description of any assumptions or corrections, such as tests of normality and adjustment for multiple comparisons
[ ]	[x]	A full description of the statistical parameters including central tendency (e.g. means) or other basic estimates (e.g. regression coefficient) AND variation (e.g. standard deviation) or associated estimates of uncertainty (e.g. confidence intervals)
[ ]	[x]	For null hypothesis testing, the test statistic (e.g. *F*, *t*, *r*) with confidence intervals, effect sizes, degrees of freedom and *P* value noted<br>*Give P values as exact values whenever suitable.*
[x]	[ ]	For Bayesian analysis, information on the choice of priors and Markov chain Monte Carlo settings
[x]	[ ]	For hierarchical and complex designs, identification of the appropriate level for tests and full reporting of outcomes
[ ]	[x]	Estimates of effect sizes (e.g. Cohen's *d*, Pearson's *r*), indicating how they were calculated

Our web collection on statistics for biologists contains articles on many of the points above.

Software and code

Policy information about availability of computer code

Data collection	Psychtoolbox-3
Data analysis	Data were preprocessed with fMRIprep 1.5.0 (Supplementary Note 2); Matlab code for ISC and ISFC analyses is available at https://github.com/BottiniLab/NarrativeArgumentative.git

For manuscripts utilizing custom algorithms or software that are central to the research but not yet described in published literature, software must be made available to editors and reviewers. We strongly encourage code deposition in a community repository (e.g. GitHub). See the Nature Research guidelines for submitting code & software for further information.

Data

Policy information about availability of data

All manuscripts must include a data availability statement. This statement should provide the following information, where applicable:

- Accession codes, unique identifiers, or web links for publicly available datasets
- A list of figures that have associated raw data
- A description of any restrictions on data availability

The datasets generated during the current study are available from the corresponding author on reasonable request. Source data underlying the figures are available in the figshare repository (doi: 10.6084/m9.figshare.14433008).

Field-specific reporting

Please select the one below that is the best fit for your research. If you are not sure, read the appropriate sections before making your selection.

- [x] Life sciences
- [ ] Behavioural & social sciences
- [ ] Ecological, evolutionary & environmental sciences

For a reference copy of the document with all sections, see nature.com/documents/nr-reporting-summary-flat.pdf

Life sciences study design

All studies must disclose on these points even when the disclosure is negative.

Sample size	We recruited 20 participants in total. Four of them were excluded (see below). This sample size was determined based on previous studies employing inter-subject correlation (Lerner et al., 2011; n = 11) and inter-subject functional connectivity (Somony et al., 2016; n = 18).
Data exclusions	Data from four participants were discarded (Supplementary Figure 13): One participant performed badly in the post-scanning questionnaire concerning the content of the narrative and argumentative texts used in the experiment (his/her accuracy was outside 1.5 times the interquartile range below the lower quartile across participants). Three participants were excluded due to excessive head motion; In two cases, the mean frame displacement index of functional images was outside 1.5 times the interquartile range above the upper quartile across participants, and one's structure image was so blurry that failed to be segmented.
Replication	We replicated the behavior rating result by using another group of participants (Supplementary Figure 1). We replicated the major findings about the brain by repeating both the intersubject correlation analysis (Supplementary Figure 6-7) and the intersubject functional connectivity analysis (Supplementary Figure 9-10) across each of the two narrative texts and across each of the two argumentative texts.
Randomization	Participants were not allocated into separate groups.
Blinding	Participants were not allocated into separate groups.

Reporting for specific materials, systems and methods

We require information from authors about some types of materials, experimental systems and methods used in many studies. Here, indicate whether each material, system or method listed is relevant to your study. If you are not sure if a list item applies to your research, read the appropriate section before selecting a response.

Materials & experimental systems

n/a	Involved in the study	
x		Antibodies
x		Eukaryotic cell lines
x		Palaeontology and archaeology
x		Animals and other organisms
	x	Human research participants
x		Clinical data
x		Dual use research of concern

Methods

n/a	Involved in the study	
x		ChIP-seq
x		Flow cytometry
	x	MRI-based neuroimaging

Human research participants

Policy information about studies involving human research participants

Population characteristics	Italian speakers who had no history of neurological or psychiatric disorders participated in the fMRI experiment. They (9 females; age range: 21 to 31, mean age: 24) were all educated (university students or above) and right-handed (laterality quotient range: +40 to +100; mean: +90).
Recruitment	We used Facebook advertising to recruit participants that met the following criteria: native Italian speakers, with no history of neurological or psychiatric disorders, right-handed, and educated (university students or above). We recruited all the participants who met these criteria without selection.
Ethics oversight	the local ethical committee at the University of Trento

Note that full information on the approval of the study protocol must also be provided in the manuscript.

Magnetic resonance imaging

Experimental design

Design type	task and resting state
Design specifications	The functional scanning included nine runs, one for the eight-minute resting state, four for the sentence-scrambled version of the texts, and four for intact version of the texts.
Behavioral performance measures	After the scanning, all participants completed a questionnaire on the content of the texts that they had heard during the scanning. We excluded one participant whose accuracy was outside 1.5 times the interquartile range below the lower quartile across participants (Supplementary Figure 13a).

Acquisition

Imaging type(s)	functional and structural
Field strength	3T
Sequence & imaging parameters	Functional images were acquired using the simultaneous multislices echoplanar imaging sequence, the scanning plane was parallel to the bicommissural plane, the phase encoding direction was from anterior to posterior, repetition time (TR) = 1000 ms, echo time (TE) = 28 ms, flip angle (FA) = 59°, field of view (FOV) = 200 mm × 200 mm, matrix size = 100 × 100, 65 axial slices, slices thickness (ST) = 2 mm, gap = 0.2 mm, voxel size = 2 × 2 × (2 + 0.2) mm, multiband factor = 5. Three-dimensional T1-weighted images were acquired using the magnetization-prepared rapid gradient-echo sequence, sagittal plane, TR = 2140 ms, TE = 2.9 ms, inversion time = 950 ms, FA = 12°, FOV = 288 mm × 288 mm, matrix size = 288 × 288, 208 continuous sagittal slices, ST = 1 mm, voxel size = 1 × 1 × 1 mm.
Area of acquisition	whole brain

Diffusion MRI ☐ Used ☒ Not used

Preprocessing

Preprocessing software	fMRIPrep 1.5.0
Normalization	A reference volume and its skull-stripped version were generated using a custom methodology of fMRIPrep. The BOLD reference was then co-registered to the T1w reference using bbregister (FreeSurfer) which implements boundary-based registration(Greve and Fischl 2009). Co-registration was configured with six degrees of freedom. Head-motion parameters with respect to the BOLD reference (transformation matrices, and six corresponding rotation and translation parameters) are estimated before any spatiotemporal filtering using mcflirt (FSL 5.0.9, Jenkinson et al. 2002). The BOLD time-series, were resampled to surfaces on the following spaces: fsaverage5.
Normalization template	fsaverage5
Noise and artifact removal	We excluded the noise induced by non-neuronal sources through two steps. First, we removed the motion-relevant noise using an Independent Component Analysis based strategy for Automatic Removal of Motion Artifacts (ICA-AROMA). The identified motion-relevant components and the signal components were fit into the same general linear model (GLM) to predict the BOLD signal in each vertex on the brain surface. We estimated the beta coefficients using the fitglm function in Matlab 2019a and subtracted the motion-relevant terms (i.e., the dot product of motion-relevant components and their estimated beta coefficients) from the BOLD signal. In this way, the motion-relevant components were removed "non-aggressively" by preserving the shared variance between the motion-relevant components and the signal components. Second, we further removed the other nuisance variables like the mean timecourses in a conservative mask of the white matter (WM) and the cerebrospinal fluid (CSF), which were extracted by fMRIPrep. As the low-frequency component (0 - 0.01 Hz) makes a significant contribution to the ISC, we did not implement high-pass temporal filtering but used the quadratic polynomial time trend to model the signal drift. Together, we fitted the WM timecourse, the CSF timecourse, and the quadratic polynomial time trend into the same GLM to predict the timecourse resulting from the first step. In the same way, we estimated the beta coefficients and subtracted the WM, the CSF, and the quadratic polynomial terms from the signal.
Volume censoring	We did not implement volume censoring.

Statistical modeling & inference

Model type and settings	For both intersubject correlation and intersubject functional connectivity analysis, each contrast's statistical likelihood was assessed using the subject-wise bootstrapping method, where the exchangeability and independence assumptions are satisfied (Chen et al., 2016). In each bootstrapping iteration, the same number of participants were randomly resampled with replacement. This procedure was repeated 5000 times to form a sampling distribution for each contrast. The null distribution of each contrast was generated by subtracting the veritable contrast value from the sampling distribution, and the veritable contrast value was then ranked against the null distribution (Hall & Wilson, 1991).
Effect(s) tested	We contrasted the intersubject correlation (ISC) and intersubject functional connectivity (ISFC) between different conditions to obtain a veritable ISC/ISFC contrast value for each contrast. The major contrasts were: (1) Scrambled Narrative Contrast: (Scrambled Narrative 1 + Scrambled Narrative 2) - 2 × Rest; (2) Intact Narrative Contrast: (Intact Narrative 1 + Intact Narrative

2) - 2 × Rest; (3) Narrative Contrast: (Intact Narrative 1 - Scrambled Narrative 1) + (Intact Narrative 2 - Scrambled Narrative 2); (4) Scrambled Argumentative Contrast: (Scrambled Argument 1 + Scrambled Argument 2) - 2 × Rest; (5) Intact Argumentative Contrast: (Intact Argument 1 + Intact Argument 2) - 2 × Rest; (6) Argumentative Contrast: (Intact Argument 1 - Scrambled Argument 1) + (Intact Argument 2 - Scrambled Argument 2); (7) Narrative Specific Contrast: [(Intact Narrative 1 - Scrambled Narrative 1) + (Intact Narrative 2 - Scrambled Narrative 2)] - [(Intact Argument 1 - Scrambled Argument 1) + (Intact Argument 2 - Scrambled Argument 2)]; (8) Argumentative Specific Contrast: [(Intact Argument 1 - Scrambled Argument 1) + (Intact Argument 2 - Scrambled Argument 2)] - [(Intact Narrative 1 - Scrambled Narrative 1) + (Intact Narrative 2 - Scrambled Narrative 2)].

Specify type of analysis: ☒ Whole brain ☐ ROI-based ☐ Both

Statistic type for inference (See Eklund et al. 2016): For intersubject correlation, vertex-wise. For intersubject functional connectivity, edge/connectivity-wise.

Correction: For intersubject correlation, FDR correction. For intersubject functional connectivity, FWE correction.

Models & analysis

n/a	Involved in the study
☐	☒ Functional and/or effective connectivity
☐	☒ Graph analysis
☒	☐ Multivariate modeling or predictive analysis

Functional and/or effective connectivity: Pearson Correlation

Graph analysis: We used the weighted graph, with the weights as the standardized effect size of each contrast. We used the node degree to measure the importance of each brain area in each contrast.
